# Novel ethyl p-methoxy cinnamate rich *Kaempferia galanga* (L.) essential oil and its pharmacological applications: special emphasis on anticholinesterase, anti-tyrosinase, α-amylase inhibitory, and genotoxic efficiencies

**DOI:** 10.7717/peerj.14606

**Published:** 2023-01-09

**Authors:** Twahira Begum, Roktim Gogoi, Neelav Sarma, Sudin Kumar Pandey, Mohan Lal

**Affiliations:** 1 Agrotechnology & Rural Development Division, CSIR-North East Institute of Science & Technology, Jorhat, Assam, India; 2 AcSIR-Academy of Scientific and Innovative Research, Ghaziabad, Uttar Pradesh, India

**Keywords:** Anti-diabetic, Antimicrobial, Essential oil, Genotoxicity, Neurodegenerative, Ethyl p-methoxycinnamate

## Abstract

**Background:**

*Kaempferia galanga* (L.) is one of the prospective therapeutic plants with an aromatic rhizome, and belongs to the Zingiberaceae family. This herb is commonly used by local practitioners in traditional Asian medicine.

**Methods:**

In the present investigation, the novel *Kaempferia galanga* rhizome essential oil rich in ethyl p-methoxy cinnamate (EMCKG) was evaluated using GC/MS for chemical composition analysis. EMCKG was analyzed for its possible antimicrobial, neurodegenerative inhibitory, acetylcholinesterase, anti-inflammatory, and antioxidant activities as well as for the genotoxic effects using the standard methodologies. ANOVA and *post hoc* was performed to test the statistical significance of the study.

**Results:**

GC/MS analysis identified ethyl p-methoxy cinnamate as the major component of EMCKG essential oil with an area percentage of 66.39%. The EMCKG exhibited moderate (DPPH assay IC_50_ = 15.64 ± 0.263 µg/mL; ABTS assay IC_50_ = 16.93 ± 0.228 µg/mL) antioxidant activity than standard ascorbic acid (DPPH assay IC_50_ = 21.24 ± 0.413 µg/mL; ABTS assay IC_50_ = 21.156 ± 0.345 µg/mL). Similarly, EMCKG showed comparable activity in albumin denaturation (IC_50_ = 2.93 ± 0.59 µg/mL) and protease inhibitor assay (IC_50_ = 17.143 ± 0.506 µg/mL) to that of standard sodium diclofenac (IC_50_ = 23.87 ± 0.729 µg/mL and IC_50_ = 19.18 ± 0.271 µg/mL, respectively). The EMCKG exhibited a dose-dependent antimicrobial activity pattern with the highest inhibitory activity at 500 µg/mL against *Staphylococcus aureus* and considerable anticholinesterase activities (IC_50_ = 21.94 ± 0.109 µg/mL) compared to the standard galanthamine (IC_50_ = 27.18 ± 0.511 µg/mL). EMCKG also showed strong anti-diabetic activity (IC_50_ = 18.503 ± 0.480 µg/mL) and anti-tyrosinase activity (IC_50_ = 14.756 ± 0.325 µg/mL) as compared to the standards used (acarbose IC_50_ = 20.39 ± 0.231 µg/mL and kojic acid IC_50_ = 17.73 ± 0.192 µg/mL) in the study. Genotoxicity analysis of EMCKG revealed that at 1 µg/mL concentration has no toxic effects in mitosis of *Allium cepa* roots (Mitotic Index MI = 13.56% and chromosomal aberration CA = 07.60%). The ANOVA confirmed that except for the anticholinesterase activity, there is insignificant difference for essential oil and standards used for all the other bioactivities thus confirming their interchangeable applicability.

**Conclusions:**

Current research provides the basis for the fact that besides being a rich source of ethyl p-methoxycinnamate, EMCKG has the potential for future formulation and development of an inexpensive skin-care agent and for the preparation of anti-diabetic drugs.

## Introduction

Medicinal and aromatic herbs have been utilized extensively for traditional medical practices since antiquity and are now used extensively in modern medicine. These pharmacologically active and aromatic plants provide raw materials for usage by various pharmaceutical companies operating in drug research (Begum, Gogoi & Lal, 2020; Gogoi, Begum & Lal, 2020). The medicinal values of the plants can be ascribed to their constituent bioactive compounds (Aiyelaagbe & Osamudiamen, 2009). *Kaempferia galanga* (L.) is a high-value aromatic medicinal plant with numerous uses from its medicinal properties point of view. It is an aromatic rhizomatous herb belonging to the Zingiberaceae family. The name of the plant was cross-checked with http://www.theplantlist.org on 1^st^ March 2021. The rhizome is tuberous with a camphoraceous odor along with some bitter aromatic taste. It is distributed widely in the tropics and sub-tropics of Asia as well as Africa (Geetha et al., 1997; Munda, Saikia & Lal, 2018). It is native to India, Thailand, Malaysia, Taiwan, and southern China of tropical Asia (Mitra, Orbell & Muralitharan, 2007; Techaprasan et al., 2010). For medicinal purposes, *K. galanga* finds its traditional use for treating headaches, inflammation, colds, asthma, hypertension, indigestion, tumor, pectoral and abdominal pains, rheumatism, and dyspepsia (Hu, 2005; Sulaiman et al., 2008; Munda, Saikia & Lal, 2018). It also finds its use in aromatherapy as well as for stress relieving, depression, anxiety and restlessness (Kanjanapothi et al., 2004; Hu, 2005; Sulaiman et al., 2008; Chan et al., 2009; Liu et al., 2010).

The economically valuable plant component of *K. galanga* is the rhizome. Due to its numerous pharmacological characteristics, the rhizome, which contains essential oil, is in high demand in the industrial sector. *K. galanga* essential oil consists of many bioactive compounds which are responsible for their various medicinal applications. The characteristics flavors and odors of essential oils are released on essential oil emission (Begum et al., 2022). Ethyl p-methoxycinnamate is one of the compounds present in *K. galanga* which possesses highly valuable medicinal properties (Munda, Saikia & Lal, 2018; Mahanta et al., 2020). The ethyl p-methoxycinnamate derived from the plant finds its wide use in medicines for its skin whiting activity, antibacterial, antiangiogenic, anti-inflammatory, anticancer and insecticidal properties and its varied use in cosmetics and food industries also (Pandji et al., 1993; Huang, Yagura & Chen, 2008; Mahanta et al., 2020). The ethyl p-methoxycinnamate compound also possesses the sedative properties of *K. galanga* by inhibiting locomotors’ activities (Umar et al., 2011). A previous study has reported that ethyl p-methoxycinnamate is capable of significantly decreasing the melanin synthesis level in α-melanocyte stimulated B16F10 murine melanoma cells (Ko et al., 2014). A copper-containing enzyme tyrosinase is one of the most important catalysts in melanin biosynthesis. The melanin accumulation in the skin is due to the activity of this enzyme. However, hyperpigmentation *i.e.*, melanin accumulation in the skin paves the way for numerous skin ailments like freckles, age spots, melasma, *etc*. (Karioti et al., 2007). The browning in foods associated with quality losses and disorder due to hyperpigmentation in human skin can be effectively controlled with therapeutic agents like tyrosinase inhibitors (Miyazawa et al., 2003). With emerging trends, a lighter skin tone is preferable further boosting the ever-growing cosmetic industries (Ko et al., 2014). For fulfilling these demand tyrosinase inhibitors finds wide application in the cosmetology industry. Plants have been searched for the fortuitous discovery of novel tyrosinase inhibitors since plants are rich, reliable sources of tyrosinase inhibitors with lesser side effects (Maisuthisakul & Gordon, 2009).

Studies have further revealed that ethyl p-methoxycinnamate possesses anti-hypertensive, anti-ulcer, and anti-inflammatory properties also. The plant also finds wide application in the cure of inflammatory conditions, lipid disorders, and diabetes mellitus (Mangaly & Sabu, 1991; Jitoe et al., 1992; Ogiso & Kobayashi, 1994; Choochote et al., 1999; Othman et al., 2002; Gogoi, Begum & Lal, 2020). Additionally, the compound has shown high cytotoxicity toward *HeLa* cells (Ridtitid et al., 2008). Another study by resazurin microtitre assay has revealed that ethyl p-methoxycinnamate is capable of inhibiting drug-susceptible multidrug-resistant strain of *Mycobacterium tuberculosis* (Lakshmanan et al., 2011). Thus, *K. galanga* with high content of ethyl p-methoxycinnamate would be highly valuable owing to its various pharmaceutical uses.

More recently the incidence of multidrug resistance has been on the rise and hence in light of this source of natural resources with effective pharmacological properties is the need of the hour. The compound ethyl p-methoxycinnamate is widely used in cosmetics (Taufikkurohmah, 2005). Moreover, it is also found to possess anti-microbial, nematicidal, anti-neoplastic mosquito-repellent and effects (Kanjanapothi et al., 2004; Kim et al., 2008; Sutthanont et al., 2010; Liu et al., 2010; Hong et al., 2011). From the pharmacological point of view, it is a strong antifungal agent as previously found in various studies. Hence, our present study is aimed at extraction of the essential oil of ethyl p-methoxycinnamate rich *K. galanga*, and analysis of its chemical composition along with evaluation for antioxidant, anti-inflammatory, antimicrobial, skin whitening ability, genotoxicity, anti-cholinesterase and anti-diabetic activity. The experiment was designed to see the effect of other compounds on ethyl-p-methoxy cinnamate pharmacological potentials. Previously high rhizome and high essential oil yielding varieties of *K. galanga* have been developed namely, ‘Jor Lab K-1’ and ‘Jor Lab Bharamaputra-1’ respectively (Lal et al., 2017; Lal, 2019a, 2019b). However, so far none of the ethyl p-methoxycinnamate compound-rich *K. galanga* is available in the public sphere. So, in the present study is the first of its kind for ethyl p-methoxycinnamate rich EMCKG along with their pharmacological properties screening for validating their industrial application. The results could be a base source for pharmaceutical products however, after proper investigation safe drugs can be developed for commercial scale.

## Materials and Methods

### Chemicals used in the study

The chemicals used were HPLC grade methanol and ethanol; potassium ferricyanide (K_3_Fe(CN)_6_); aluminum chloride (AlCl_3_); tris-buffer (C_4_H_11_NO_3_); ethyl methanesulfonate (C_3_H_8_O_3_S); ascorbic acid (C_6_H_8_O_6_); sodium carbonate (Na_2_CO_3_); ferric chloride (FeCl_3_); acetocarmine (CH_3_CO_2_H); ethylene diamine tetraacetic acid (EDTA) (C_10_H_16_N_2_O_8_); 2,2-diphenyl 1 picrylhydrazyl (C_18_H_12_N_5_O_6_); potassium persulfate (K_2_S_2_O_8_); MHA-Mueller Hinton Agar and PDA-Potato Dextrose agar were procured from HiMedia (India). ABTS (2, 2′-azino-bis (3-ethylbenzothiazoline-6-sulfonic acid) (C_18_H_18_N_4_O_6_S_4_); trichloroacetic acid (C_2_HCl_3_O_2_); ferrous chloride (FeCl_2_), ferrozine (C_20_H_12_N_4_Na_2_O_6_S_2_) and diclofenac sodium salt (C_14_H_10_Cl_2_NNaO_2_) were purchased from Sigma Aldrich (Germany). A fresh egg of a hen used for albumin and all the other unlabeled chemicals were obtained from the market of Jorhat, Assam India.

### Plant material collection and extraction of essential oil

Ethyl p-methoxycinnamate rich *K. galanga* L. (cv. Jor Lab K-2) is cultivated at the experimental farm of CSIR-NEIST, Jorhat, India (26°44′00.0″ N, 94°09′50.0″ E) under the supervision of the plant breeder Dr. Mohan Lal, CSIR-NEIST Jorhat. A voucher specimen was placed in the CSIR-NEIST Jorhat departmental herbarium vide herbarium no RRLJKN-17. For the essential oil isolation matured rhizomes of *K. galanga* (*cv*. Jor Lab K-2) were harvested during December 2020. Collected rhizomes were washed, to remove the sand particles which were cut into smaller slices. The sliced rhizomes were shade dried for 7 days followed by oven drying at the temperature of 70 °C for 3 days until a constant mass was obtained. The essential oil was isolated from dried rhizomes using the Clevenger apparatus as per Lal (2019a).

### GC/MS analysis of *Kaempferia galanga* essential oil

The GC/MS analysis of EMCKG was done as per Begum et al. (2022).

**Table:** 

GC/MS	Agilent technologies gas chromatograph
Column	HP-5MS (30 m × 0.25 mm i.d.; film thickness 0.25 μm)
Detector	MSD 5975 C
Carrier gas	Helium (1 mL/min flow rate)
Temperature range	40 °C (2 min) followed by 25 °C with a rate of 5 °C/min and lastly 300 °C at 30 °C/min (10 min)
Scanning range	45 to 650 amu

### Protein (egg albumin) denaturation assay

The EMCKG was evaluated for anti-inflammatory activity as per Gogoi et al. (2020a). The EMCKG was analyzed at a varying concentration ranging from 50 to 300 µg/mL for anti-inflammatory activity. IC_50_ *i.e.*, 50% inhibition was analyzed using MS Excel.

### Protease inhibitor activity

Protease inhibitor activity was analyzed using a standard method as per Gogoi et al. (2020b). For the analysis, EMCKG was prepared with different concentrations which ranged from 1.5 to 48 µg/mL. IC_50_ *i.e.*, 50% inhibition was analyzed using MS Excel.

### Antimicrobial activities

#### Microbial strains used

The EMCKG antimicrobial activities were analyzed using both gram-positive and gram-negative bacteria. The gram-positive bacteria used were *Staphylococcus aureus, Micrococcus luteus, Streptococcus mutans, Bacillus subtilis* and *Bacillus cereus* with ATCC-11632, ATCC-10240, ATCC-25175, ATCC-11774 and ATCC-10876, respectively. *Salmonella typhimurium and Salmonella enteric* with ATCC-13311, and ATCC-35664 were used as gram-negative bacteria. In addition to those four fungal strains namely, *Aspergillus niger, Fusarium keratoplasticum, Aspergillus oryzae, Saccharomyces cerevisiae, Aspergillus fumigatus*, and *Candida albicans* with ATCC-16885, ATCC-36031, ATCC-10124, ATCC-9763, ATCC-204305, ATCC-66027 respectively were employed for evaluation of the antifungal activity. Standard Ciprofloxacin (antibacterial) and Fluconazole (antifungal) each at 10 µg/disc were used as standard drugs.

#### DD and MIC assays

The EMCKG was analyzed for their antimicrobial activity as per Pandey et al. (2022). The EMCKG was analyzed for their antimicrobial activity by agar disc diffusion method at various concentrations ranging from 50 to 500 μg/mL. The minimal inhibitory concentration (MIC) was analyzed using the method of broth microdilution.

#### Growth curve study

The bacterial growth curve experiment was performed to analyze the bactericidal capabilities of EMCKG essential oil. The experiment was performed as per the protocol of Huang et al. (2018) with slight modifications. The bacteria were treated with different concentrations of the essential oil *viz*., 50, 100, 250 and 500 μg/mL. The broth was taken as blank with a control that is free from the essential oil. Data were recorded at an interval of 1 h continuous up to 8 h. The measurements of the optical density (OD_600_) were taken using the Eppendorf Bio spectrometer basic. Time was taken along the horizontal axis and the OD of the supernatants was taken against the vertical axis.

#### Acetylcholinesterase activity assay

The EMCKG was analyzed for acetylcholinesterase inhibitory (AChE) activity using a standard modified methodology as per Begum et al. (2022). The EMCKG in the amount of 50 µL was added for the reaction analysis. IC_50_ *i.e.*, 50% inhibition was analyzed using MS Excel.

#### Anti-diabetic analysis

The antidiabetic activity of EMCKG was analyzed using standard methodology as per Begum et al. (2022). For the evaluation, the EMCKG was prepared in various concentrations ranging from 5 to 30 µg/mL. IC_50_ *i.e.*, 50% inhibition was analyzed using MS Excel.

#### Anti-tyrosinase activity assay

Tyrosinase inhibitory assay was conducted as per Begum et al. (2022). For the analysis, 125 µL of EMCKG was used in the reaction involved. IC_50_ *i.e.*, 50% inhibition was analyzed using MS Excel.

### Antioxidant activity

#### DPPH assay

DPPH free radical scavenging activity of EMCKG was performed as per Gogoi et al. (2020a). The EMCKG was used at a concentration ranging from 5 to 30 μg/mL. IC_50_ *i.e*., 50% inhibition was analyzed using MS Excel.

#### ABTS assay

The ABTS assay was performed following the protocol as per Dutta et al. (2021). The EMCKG at various concentrations ranging from 10–60 µg/mL was used for the analysis. IC_50_ *i.e.*, 50% inhibition was analyzed using MS Excel.

#### Metal chelating activity assay

The metal chelating activity assay was conducted for the antioxidant activity of EMCKG as per Begum et al. (2022). The EMCKG at various concentrations ranging from 10–60 µg/mL was used for the analysis. IC_50_ *i.e.*, 50% inhibition was analyzed using MS Excel.

#### Reducing antioxidant power determination

The reducing power of EMCKG was evaluated using a standard protocol as mentioned by Gogoi et al. (2020b). The EMCKG at various concentrations ranging from 5–30 µg/mL was used for the analysis. IC_50_ *i.e.*, 50% inhibition was analyzed using MS Excel.

#### Test for genotoxicity (*Allium cepa* assay)

The genotoxicity of EMCKG was analyzed using a standard method as per Begum et al. (2022). The EMCKG at 1 µg/mL concentration was used for the study.

### Statistical analysis

The standard deviation (SD) was analyzed using MS Excel. Each of the experiments was performed in triplicates to reduce experimental errors (when *p* ≤ 0.05 the difference was considered statistically significant). The ANOVA analysis was done followed by a *post hoc* analysis.

## Results

### GC/MS analysis of *K. galanga* essential oil

The GC/MS analysis report of the EMCKG revealed ethyl p-methoxy cinnamate (66.39%) as the primary major compound followed by *trans*, ethyl cinnamate (9.86%) and 3-carene (9.60%). The other compounds present were in minor (<5%) amounts. The minor compounds present were endo-borneol (2.76%), camphene (1.66%), α-pinene (1.68%), eucalyptol (1.24%), benzyl benzoate (0.49%), *p-*cymene (1.12%), D-limonene (1.06%), pentadecane (1.00%), *cis*-p-mentha-2,8-dien-1-ol (0.80%), and bornyl acetate (0.72%) (Table 1). The cinnamic acid esters composed a majority of the component of EMCKG constituting 76.25% of the total identified compounds followed by monoterpene hydrocarbon (15.12%), oxygenated monoterpene (4.00%), sesquiterpene hydrocarbon (1.00%), monoterpenoid (0.72%) and lastly benzoate ester (0.49%).

**Table 1:** GC/MS analysis of essential oil of ethyl p-methoxy cinnamate rich *Kaempferia galanga* essential oil (MCKG).

Sl. no.	Name of the compound	RT	Area %	RI*	RI**	Identification method
1	α-Pinene	5.77	1.68	937	940	1,2,3
2	Camphene	6.05	1.66	952	954	1,2,3
3	3-Carene	7.18	9.60	1,011	1,006	1,2,3
4	p-Cymene	7.44	1.12	1,025	1,022	1,2
5	D-Limonene	7.52	1.06	1,030	1,028	1,2,3
6	Eucalyptol	7.58	1.24	1,032	1,033	1,2,3
7	*cis*-p-Mentha-2,8-dien-1-ol	8.84	0.80	1,122	1,123	1,2
8	endo-Borneol	10.12	2.76	1,167	1,064	1,2
9	Bornyl acetate	12.26	0.72	1,285	1,283	1,2
10	*trans*, Ethyl cinnamate	15.18	9.86	1,464	1,462	1,2,3
11	Pentadecane	15.63	1.00	1,500	1,497	1,2,3
12	Ethyl p-methoxycinnamate	19.40	66.39	1,785	1,781	1,2,3
13	Benzyl Benzoate	19.57	0.49	1,762	1,763	1,2
Total = 100%, Identified compounds = 98.38%, Unidentified compounds = 1.62%						
Monoterpene hydrocarbons (Sl No. 1–5) = 15.12%						
Oxygenated monoterpene (Sl No. 6, 8) = 4.00%						
Monoterpenoid (Sl No. 9) = 0.72%						
Sesquiterpenes hydrocarbons (Sl No. 11) = 1.00%						
Cinnamic acid esters (Sl No. 10, 12) = 76.25%						
Benzoate ester (Sl No. 13) = 0.49%						
Others (Sl No. 7) = 0.80%						

**Note:**

RI*, Retention Index Literature (Goodner, 2007; Kovats, 1965); RI**, Retention Index Experimental; RT, Retention time; MCKG, ethyl p-methoxy cinnamate rich *Kaempferia galanga* essential oil, 1. Comparison of retention indices with literatures, 2. Comparison of the mass spectra with the mass libraries, 3. Comparing retention time with standards injected with same GC condition.

### Anti-inflammatory activity

The EMCKG was analyzed for anti-inflammatory activity using two different methods albumin denaturation assay and protease inhibitor activity assay. The EMCKG showed anti-inflammatory activity with an IC_50_ value of 2.93 µg/mL as compared to that of standard sodium diclofenac 19.18 µg/mL for protein denaturation assay, while protease inhibitor assay revealed IC_50_ of 17.14 µg/mL for EMCKG and 23.87 µg/mL for standard sodium diclofenac (Table 2).

**Table 2:** 50% Inhibition concentrations (IC_50_) determination values for pharmacological activities of MCKG and standards using MS-EXCEL software.

Essential oil/ Standard	DPPH scavenging (µg/mL)	ABTS scavenging (µg/mL)	Metal chelating (µg/mL)	Protein denaturation (µg/mL)	Protease inhibitory (µg/mL)	Tyrosinase inhibitory (µg/mL)	Acetylcholinesterase inhibitory (µg/mL)	α-Amylase inhibitory (µg/mL)
MCKG	15.64 ± 0.263	16.93 ± 0.228	19.29 ± 0.805	2.93 ± 0.59	17.143 ± 0.506	14.756 ± 0.325	21.94 ± 0.109	18.503 ± 0.480
Ascorbic acid	21.24 ± 0.413	21.156 ± 0.345	nd	nd	nd	nd	nd	nd
EDTA	nd	nd	30.72 ± 0.834	nd	nd	nd	nd	nd
Sodium diclofenac	nd	nd	nd	19.18 ± 0.271	23.87 ± 0.729	nd	nd	nd
Kojic acid	nd	nd	nd	nd	nd	17.73 ± 0.192	nd	nd
Galanthamine hydrobromide	nd	nd	nd	nd	nd	nd	27.18 ± 0.511	nd
Acarbose	nd	nd	nd	nd	nd	nd	nd	20.39 ± 0.231

**Note:**

µg, microgram; mL, millilitre; SD, standard deviation; IC_50_, 50% of Inhibition concentration; %, percentage; MCKG, ethyl p-methoxycinnamate rich *K. galanga* essential oil; nd, not determined.

### Antimicrobial activity

The study reveals that the *K. galanga* essential oil exhibited antimicrobial activity in a concentration-dependent manner with the highest inhibition zone at 500 µg/mL while the lowest activity was at 50 µg/mL (Table 3). The MIC revealed that *K. galanga* essential oil exhibited prominent antibacterial activity as compared to antifungal activity (Table 4).

**Table 3:** Antimicrobial activity of ethyl p-methoxy cinnamate rich *K. galanga* essential oil.

Name of the essential oil	Bacteria/Fungus	Inhibition zone (mm)				Standard diameter in mm (ciprofloxacin/Fluconazol, 10 µg/mL)
		50 µg/mL	100 µg/mL	250 µg/mL	500 µg/mL	
MCKG	*S. aureus*	13 ± 0.58	20 ± 1.5	27 ± 1.2	35 ± 1.5	18 ± 1
	*B. subtilis*	10 ± 1	13 ± 1.52	25 ± 0.6	31 ± 1.52	38 ± 1.53
	*B. cereus*	14 ± 1.53	15 ± 2.08	20 ± 1.2	26 ± 1.15	15 ± 1.52
	*S. typhimurium*	12 ± 1.15	15 ± 1.52	19 ± 1	20 ± 1.52	19 ± 0.58
	*M. luteus*	8 ± 0.5	12 ± 0.5	18 ± 0.5	22 ± 0.5	44 ± 1.15
	*S. mutans*	8 ± 0.5	9 ± 0.5	11 ± 0.5	14 ± 1	25 ± 1
	*S. enterica*	10 ± 0.5	11 ± 0.5	12 ± 0.5	14 ± 0.5	30 ± 1.20
	*S. cereviaceae*	17 ± 0.5	19 ± 0.5	21 ± 0.5	24 ± 0.5	27 ± 0.5
	*C. albicans*	11 ± 0.5	15 ± 0.5	17 ± 0.5	21 ± 0.5	29 ± 0.5
	*A. oryzae*	7 ± 0.5	9 ± 0.5	16 ± 0.5	19 ± 0.5	10 ± 1.25
	*F. keratoplasticum*	NA	10 ± 0.5	12 ± 0.5	16 ± 0.5	28 ± 0.50
	*A. fumigatus*	NA	NA	11 ± 0.5	13 ± 0.5	21 ± 0.5
	*A. niger*	NA	NA	NA	10 ± 0.5	26 ± 0.47

**Note:**

µg, microgram; mL, millilitre; MCKG, ethyl p-methoxycinnamate rich *K. galanga* essential oil.

**Table 4:** Minimum inhibitory concentration (MIC) for ethyl p-methoxy cinnamate rich *K. galanga* essential oil.

Microbial strains	MIC values (μg/mL) MCKG	MIC values (μg/mL) ciprofloxacin	MIC values (μg/mL) fluconazole
*B. cereus*	48.00	03.50	*NA*
*S. aureus*	12.50	08.20	*NA*
*M. luteus*	10.50	12.50	*NA*
*B. subtilis*	8.50	12.15	*NA*
*S. typhimurium*	8.50	10.25	*NA*
*S. enterica*	8.25	15.25	*NA*
*S. mutans*	5.25	08.50	*NA*
*A. oryzae*	15.50	*NA*	12.25
*A. fumigatus*	10.25	*NA*	15.55
*S. cereviaceae*	8.50	*NA*	10.25
*A. niger*	6.75	*NA*	15.50
*C. albicans*	6.25	*NA*	08.10
*F. keratoplasticum*	6.25	*NA*	15.00

**Note:**

µg, microgram; mL, millilitre; MCKG, ethyl p-methoxycinnamate rich *K. galanga* essential oil.

**Figure 1:**
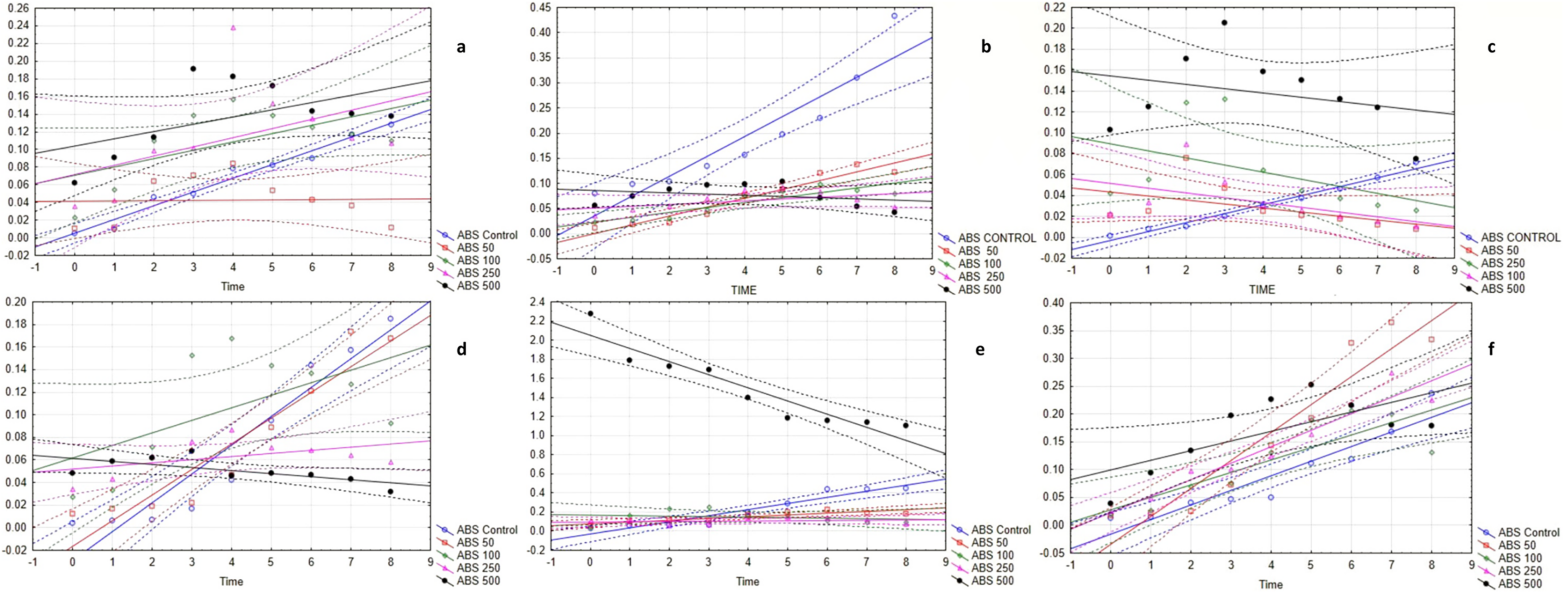
The bacterial growth curves affected by ethyl p-methoxycinnamate rich *K. galanga* essential oil. (A) *B. subtilis*, Scatterplot of multiple variables against Time. ?????1 in *B. subtilis* growth curve KN 17 6v*9c; ABS Control = 0.0051 + 0.0155*×; 0.95 Conf. Int.; ABS 50 = 0.0419 + 0.0003*×; 0.95 Conf. Int.; ABS 100 = 0.0706 + 0.0095*×; 0.95 Conf. Int.; ABS 250 = 0.072 + 0.0104*× 0.95 Conf. Int.; ABS 500 = 0.1044 + 0.0082*×; 0.95 Conf. Int. (B) *S. mutans*, Scatterplot of multiple variables against Time. ?????1 in *S. mutans* growth curve KN 17 6v*9c; ABS Control = 0.0359 + 0.0394*×; 0.95 Conf. Int.; ABS 50 = 0.0005 + 0.0175*×; 0.95 Conf. Int; ABS 100 = 0.0227 + 0.0097*×; 0.95 Conf. Int.; ABS 250 = 0.051 + 0.0035*× 0.95 Conf. Int.; ABS 500 = 0.0854 − 0.0024*×; 0.95 Conf. Int. (C) *S. aureus*, Scatterplot of multiple variables against Time. ?????1 in *S. aureus* growth curve KN 17 6v*9c; ABS Control = −0.0034 + 0.0087*×; 0.95 Conf. Int.; ABS 50 = 0.0436 − 0.0039*×; 0.95 Conf. Int.; ABS 100 = 0.0513 − 0.0045*×; 0.95 Conf. Int.; ABS 250 = 0.0895 − 0.0068*× 0.95 Conf. Int.; ABS 500 = 0.1548 − 0.0041*×; 0.95 Conf. Int. (D) *M. luteus*, Scatterplot of multiple variables against Time. ?????1 in M. luteus growth curve KN 17 6v*9c; ABS Control = −0.0289 + 0.0255*×; 0.95 Conf. Int.; ABS 50 = −0.0167 + 0.0228*×; 0.95 Conf. Int.; ABS 100 = 0.0615 + 0.0111*×; 0.95 Conf. Int.; ABS 250 = 0.0517 + 0.0028*× 0.95 Conf. Int.; ABS 500 = 0.0611 − 0.0027*×; 0.95 Conf. Int. (E) *B. cereus*, Scatterplot of multiple variables against Time. ?????1 in *B. cereus* growth curve KN 17 6v*9c; ABS Control = −0.0278 + 0.0633*×; 0.95 Conf. Int.; ABS 50 = 0.0784 + 0.0182*×; 0.95 Conf. Int.; ABS 100 = 0.1701 - 0.0055*×; 0.95 Conf. Int.; ABS 250 = 0.0951 + 0.0029*× 0.95 Conf. Int.; ABS 500 = 2.0464 − 0.1376*×; 0.95 Conf. Int. (F) *S. typhimurium*, Scatterplot of multiple variables against Time. ?????1 in *S. typhimurium* growth curve KN 17 6v*9c; ABS Control = −0.0165 + 0.0264*×; 0.95 Conf. Int.; ABS 50 = −0.034 + 0.0502*×; 0.95 Conf. Int.; ABS 100 = 0.0272 + 0.0225*×; 0.95 Conf. Int.; ABS 250 = 0.0232 + 0.0296*× 0.95 Conf. Int.; ABS 500 = 0.0996 + 0.0173*×; 0.95 Conf. Int.

The bacterial growth curve assay further revealed the potent antimicrobial activity of EMCKG. The experiment showed that the growth curve of the control increases with time, however, the results were different in the essential oil treatments. In the case of the addition of ethyl p-methoxy cinnamate-rich *K. galanga* essential oil at different concentrations, the growth of the bacteria took a longer time or in higher concentrations, its growth stopped which means the essential oil hampered the lag and log phase. It is seen that the essential oil is effective towards all the bacteria in high concentrations and in lower concentrations it somehow affects the growth of the bacteria which is in the same line with the other antimicrobial activity performed (Fig. 1).

### Anti-cholinesterase activity

The anticholinesterase activity of the *K. galanga* essential oil was analyzed using standard methodology. For the assay, the IC_50_ value for standard galanthamine was 27.18 µg/mL whereas the essential oil sample showed an IC_50_ value of 21.94 µg/mL as analyzed by MS-Excel (Table 2).

### Anti-diabetic activity

Ethyl p-methoxy cinnamate-rich *K. galanga* essential oil was analyzed for its anti-diabetic activity using α-amylase inhibitory activity assay. The IC_50_ value for EMCKG was found to be 18.50 µg/mL while for the standard it was found to be 20.39 µg/mL (Table 2).

### Anti-tyrosinase activity

Skin whitening or anti-tyrosinase activity was analyzed using a standard tyrosinase inhibitor assay. *K. galanga* essential oil IC_50_ 14.75 µg/mL emerged as a promising source of skin whitening agent better than standard Kojic acid IC_50_ 17.73 µg/mL (Table 2).

### Antioxidant activity

**Figure 2:**
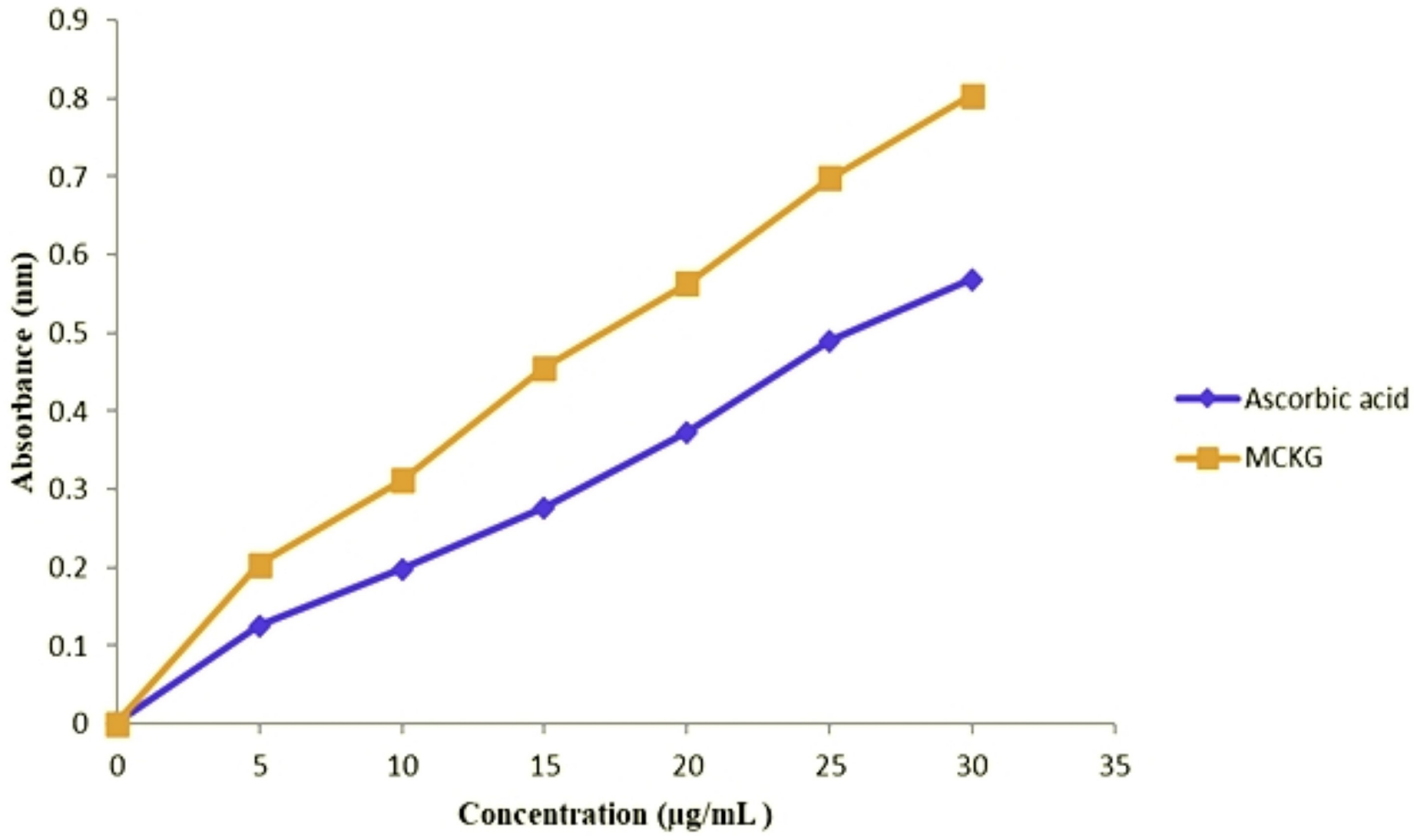
Reducing power activities of ethyl p-methoxycinnamate rich *Kaempferia galanga* essential oiland ascorbic acid.

The EMCKG showed antioxidant activity with an IC_50_ value of 15.64 µg/mL as compared to that of standard ascorbic acid 21.24 µg/mL for DPPH assay, 16.93 µg/mL for EMCKG while 21.15 µg/mL for standard ascorbic acid for ABTS assay and 19.29 µg/mL for EMCKG and 30.72 µg/mL for EDTA standard for metal chelating assay (Table 2). The reducing power determination assay revealed that EMCKG exhibited better antioxidant properties than the standard ascorbic acid. The antioxidant activity was found to be dose-dependent and showed maximum activity at a concentration of 30 µg/mL (Fig. 2).

### Genotoxicity activity

**Figure 3:**
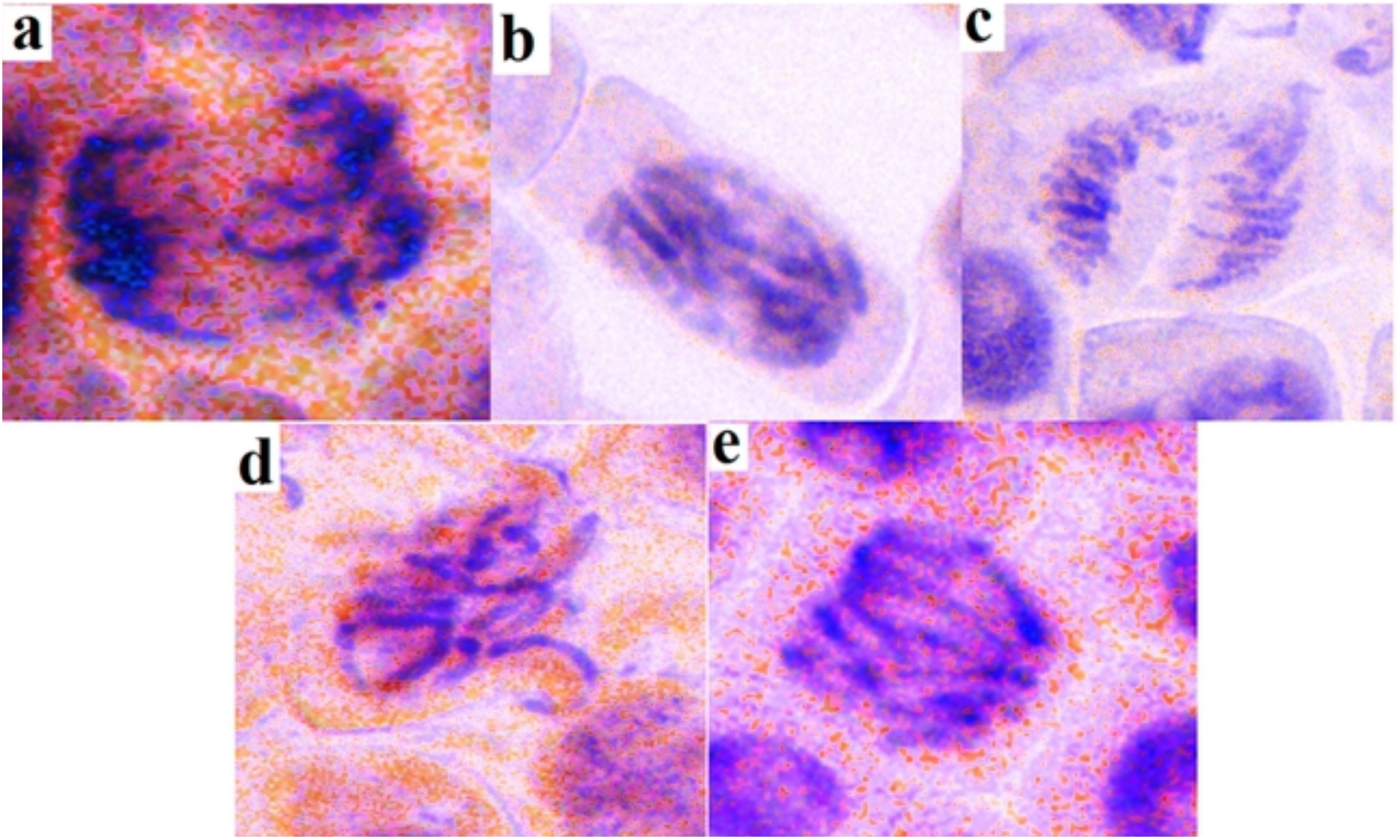
Ethyl p-methoxycinnamate rich *K. galanga* essential oil genotoxicity chromosomal aberrations. (A) Chromosome breakage, (B) chromosome bridge, (C) clumped chromosome, (D) sticky chromosomes, and (E) multi-polarity.

The genotoxicity of *K. galanga* essential oil was analyzed using an *Allium cepa* assay. Root growth test showed 0.77, 0.29, and 0.07 cm of total growth after 72 h of treatment with distilled water, *K. galanga* essential oil, and ethyl methanesulfonate respectively (Table 5). The mitotic index value of *K. galanga* essential oil 13.56% again showed as very reliable and non-genotoxic in 1 µg/mL concentration close to distilled water 19.20% (Table 6). The chromosome aberration test for *K. galanga* essential oil showed an aberration rate of 07.60%, which was very close to distilled water 05.60% but far from EMS 16.80% (Table 7, Fig. 3).

**Table 5:** *Allium cepa* root growth test for ethyl p-methoxy cinnamate rich MCKG.

Sample/standard (1 µg/mL)	Root lengths before 72 h of treatment (cm) ± SD	Root lengths after 72 h of treatment (cm) ± SD	Total root growth in 72 h (cm)
EMS	07.72 ± 0.012	07.79 ± 0.001	0.07
Distilled H_2_O	06.14 ± 0.044	06.91 ± 0.012	0.77
MCKG	06.18 ± 0.042	06.47 ± 0.034	0.29

**Note:**

µg, microgram; mL, millilitre; cm, centimetre; EMS, ethyl methanesulfonate; MCKG, ethyl p-methoxy cinnamate rich *K. galanga* essential oil.

**Table 6:** Mitotic index calculation for ethyl p-methoxy cinnamate rich *K. galanga* essential oil.

Sample/Standard (1 µg/mL)	MI (%)	Prophase	Metaphase	Anaphase	Telophase
EMS	03.82%	91.43%	08.57%	00.00%	00.00%
Distilled H_2_O	19.20%	44.92%	30.57%	18.24%	06.27%
MCKG	13.56%	56.38%	28.06%	11.52%	04.04%

**Note:**

µg, microgram; mL, millilitre; MI, mitotic index; EMS, ethyl methanesulfonate; MCKG, ethyl p-methoxycinnamate rich *K. galanga* essential oil.

**Table 7:** Chromosome aberration test for ethyl p-methoxy cinnamate rich MCKG.

Sample/standard (1 µg/mL)	CA (%)	CB	CS	CC	CBr	Mu
EMS	16.80%	28	17	19	8	12
Distilled H_2_O	05.60%	5	4	3	10	6
MCKG	07.60%	12	5	7	6	8

**Note:**

µg, microgram; mL, millilitre; CA, Chromosome aberration; CB, Chromosome Bridge; CS, Sticky Chromosome; CC, Clumped Chromosome; CBr, Chromosome Breakage; Mu, Multipolarity; EMS, ethyl methanesulfonate; MCKG, ethyl p-methoxycinnamate rich *K. galanga* essential oil.

The analysis of variance was enumerated for all the bioactivities for both standards used and the *K. galanga* essential oil (Tables S1.1–S1.7 and S2). The ANOVA reveals the presence of statistically significant differences only for the anticholinesterase activity (Table S1.5). All the other bioactivities were statistically insignificant revealing that the essential oil analyzed and the standards used are interchangeable. The *post hoc* analysis using Tukey HSD test was performed which further confirms the significant differences between standard used and the *K. galanga* essential oil for acetylcholinesterase activity (Table S2). Thus, the *K. galanga* essential oil is interchangeable with the standards used for all the bioactivities except for the anticholinesterase activity.

## Discussion

### GC/MS analysis of *K. galanga* essential oil

The GC/MS analysis of the present study on EMCKG was ethyl p-methoxy cinnamate rich constituting 66.39% of the total area percentage. As per a previous study on the EMCKG of the identified compounds the major components identified were ethyl *trans*-cinnamate (31.12%) followed by ethyl *trans*-p-methoxycinnamate (14.3%), eucalyptol (10.57%), δ-3-carene (5.12%), and n-pentadecane (4.8%), respectively (Lal et al., 2017). While in another study conducted the major compounds were found to be propanoic acid, pentadecane, and ethyl-p-methoxycinnamate. The other compounds present in minor amounts were isopropyl cinnamate, 1,8-cineol, dicyclohexyl propanedinitrile, ethyl cyclohexyl acetate, undecanone, 9-hydroxy, 2,7-octadiene-1-yl acetate, 2-nonanone, 4-methyl isopulegone, dipentene dioxide, camphene, *cis*-11-tetradecenyl acetate, camphidine, α-pinene, caryophyllenes, 3,7-dimethoxycoumarin, δ-3-carene, cadinenes, α-terpineol, germacrenes, α-gurjunene, 10 undecyn-1ol, luteolin and apigenin (Khare, 2004; Othman et al., 2006; Koh, 2009; Sutthanont et al., 2010).

Similar results were obtained in a study where the major compounds present were ethyl-p-methoxycinnamate, 1-8-cineole, carvone, methyl cinnamate, pentadecane, borneol, camphene, benzene, and α-pinene present in 31.77%, 23.23%, 11.13%, 9.59%, 6.41%, 2.87%, 2.47%, 1.33% and 1.28% respectively (Tewtrakul et al., 2005). A recent report by Mahanta et al. (2020), reported ethyl p-methoxycinnamate as the major compound of *K. galanga* essential oil constituting 61.6% of the essential oil. The previous report is in a similar line to our investigations. However, another report revealed that β-phellandrene followed by α-terpineol, ethyl cinnamate and dihydro β-sesquiphellandrene was the major compounds of *K. galanga* essential oil (Sudibyo, 2000). The present study revealed that the present *K*. *galanga* essential oil is rich in ethyl p-methoxycinnamate which has great industrial applications and could be used for the different pharmaceutical purposes as well as separate the fraction of this important compound as a chip source of ethyl *trans*-p-methoxycinnamate.

### Anti-inflammatory activity

The present analysis of EMCKG for anti-inflammatory activity revealed potent anti-inflammatory properties of EMCKG which was found to be stronger than that of standard. A previous report on the *K. galanga* leaf essential oil revealed that it possesses a dose-dependent anti-inflammatory effect in rats in concentrations of 30, 100 and 300 mg/kg respectively (Sulaiman et al., 2008). Similarly, another report revealed that the *K. galanga* leaf essential oil displays significant inflammation inhibitory properties in rats at the concentration of 30, 100, and 300 mg/kg (Tewtrakul et al., 2005). *K. galanga*, extracts were also reported to inhibit neutrophil infiltration thereby suppressing the progression of chronic and acute inflammation in rats (Jagadish et al., 2016).

A study on the ethyl-p-methoxycinnamate for its anti-inflammatory effect was found to inhibit carrageenan-induced edema in a dose-dependent manner with a minimum inhibitory concentration (MIC) value of 10 mg/kg (Umar et al., 2011). A previous report on *in vitro* assay of ethyl-*p*-methoxycinnamate reported inhibition of cyclooxygenase enzymes 1 (COX-1) (42.9%) and 2 (COX-2) (57.82%) whereas the standard indomethacin similarly inhibited COX-1 (82.8%) and COX-2 (54.6%) (Umar et al., 2012). It was concluded that significant anti-inflammatory potential exhibited by ethyl-*p*-methoxycinnamate was done by the inhibition of angiogenesis, and pro-inflammatory cytokines in return which inhibited the functioning of endothelial cells. The study revealed that ethyl-*p*-methoxycinnamate could be an effective source as a therapeutic agent for the treatment of inflammatory-related disorders (Umar et al., 2014).

In another study, the petroleum ether, a crude alcoholic extract of *K. galanga* was found to have the maximum quantity of ethyl-*p*-methoxycinnamate. Against acute inflammation, in rats, petroleum ether extract was found effective at 300 mg/kg concentration. While petroleum ether extract at a concentration of 100 mg/kg was found to effectively reverse the inflammation (Jagadish et al., 2016). In a study that reported the alcoholic extract the anti-inflammatory activity of *K. galanga* was analyzed in animal models. In the study, two doses of plant extract exhibited significant inflammation-reducing capacity in the cotton pellet granuloma model and carrageenan model in 600 and 1,200 mg/kg doses (Vittalrao et al., 2011). When the aqueous extracts of *K. galanga* leaves were given subcutaneously they displayed significant inflammation-inhibitory properties in rats in a dose-dependent manner (Sulaiman et al., 2008). The presence of high ethyl p-methoxycinnamate content can be attributed as the reason for the significant anti-inflammatory activity as various previous studies have also shown its anti-inflammatory activities. Hence the study reveals the high anti-inflammatory activity of EMCKG which can be potentially used and further validates the application of *K. galanga* in ethnomedicine for its anti-inflammatory properties.

### Antimicrobial activity

EMCKG was found to exhibit antimicrobial activity in a concentration-dependent manner. While the MIC and growth curve experiment revealed better antibacterial properties than antifungal properties of EMCKG. Previous studies revealed that extracts of *K. galanga* from which ethyl-p-methoxycinnamate was isolated have significant activities against *Candida albicans* and *Mycobacterium tuberculosis* (Kanjanapothi et al., 2004; Techaprasan et al., 2010). Moreover, resazurin microtitre assay of ethyl-p-methoxycinnamate revealed its capacity to inhibit multidrug-resistant and drug-susceptible strains of *M. tuberculosis* (MIC 0.242 to 0.485 mM) (Lakshmanan et al., 2011). Another study revealed the antimicrobial activity of *K. galanga* extracts against selected microbial strains of *Escherichia coli, Klebsiella pneumonia, Serratia marcescens, Pseudomonas aeruginosa*, *Staphylococcus aureus, Streptococcus pyogenes, Vibrios parahaemolyticus, Candida albicans, Vibrios cholera, Enterococcus faecalis* and *Salmonella typhi* (Mekseepralard, Kamkaen & Wilinson, 2010). *Kaempferia galanga* rhizome extract ethyl-p-methoxy cinnamate was screened for its antibacterial activity which revealed that concentrations up to 1.2% and 2.4% have a minimum inhibitory concentration (MIC) against *S. epidermidis* and *S. aureus*; while for *P. acne*, the concentrations are 0.6%, 1.2%, and 2.4% (Dash, Nasrin & Ali, 2014). Another study reported that the aqueous extracts, methanol, ethanol, chloroform and petroleum ether of *K. galanga* possess microbial growth inhibition against *Enterobacter aerogenes, Staphylococcus aureus, Escherichia coli, Bacillus subtilis, Salmonella typhimurium, Bacillus cereus, Streptococcus faecalis, Pseudomonas aeruginosa, Candida albicans, Klebsiella pneumoniae, Vibrio cholera*, *Aspergillus niger, A. fumigatus*, and *A. flavus* (Elya et al., 2016). However, Samsol et al. (2021), reported that *Kaempferia galanga* essential oil exhibited low activity against *Bacillus subtilis, Bacillus cereus, Klebsiella pneumoniae, Aspergillus spp and Saccharomyces cerevisiae*.

Tewtrakul et al. (2005), reported that the EMCKG exhibited marked antimicrobial activity against *C. albicans*, gram-negative, positive bacteria with the zone of inhibition of 31.0, 8.0–12.0 and 12.0–16.0 mm respectively. It was found to be stronger than that of standard antifungal Clotrimazole which had an inhibition zone of 25.0 mm against *C. albicans* (Tewtrakul et al., 2005). The present findings thus revealed that EMCKG possesses more potent antimicrobial activity which is comparable to that of the standards used in the study. EMCKG thus has the scope to be used as an antimicrobial agent with better potential as an antibacterial agent as compared to the antifungal potential.

### Anti-cholinesterase activity

Anticholinesterase activity revealed that EMCKG was comparable to that of standard. So far, the acetylcholinesterase activity for *K. galanga* essential oil has not been studied. However, a study on the methanolic extract of *K. parviflora* as well as its compounds was conducted which revealed that it inhibited butyryl cholinesterase (BChE) and acetylcholinesterase (AChE). This study is significant in Alzheimer’s disease treatment (Sawasdee et al., 2009). Thus, EMCKG possesses potential to be used for the acetylcholinesterase property with further evaluation.

### Anti-diabetic activity

The present study on the anti-diabetic activity of EMCKG revealed EMCKG possess potent anti-diabetic property and surpassed the standard. There were few studies reported as *K. galanga* rhizome extracts possess anti-diabetic effects that reduce the blood cholesterol level in rats (Sudatri et al., 2019). The mentioned investigation reported that the decrease in blood cholesterol may be due to the presence of flavonoids. In the present investigation, the anti-diabetic potential of *K. galanga* essential oil could be positively correlated with the presence of triterpenoids, steroids, *etc*. (Mohammad et al., 2006). Because triterpenes appear to exhibit sufficient characteristics. Numerous studies have demonstrated that these substances have various anti-diabetic actions. They can normalize plasma glucose and insulin levels, stop the onset of insulin resistance and inhibit enzymes involved in glucose metabolism (Nazaruk & Borzym-Kluczyk, 2015). Additionally, α-amylase is a primary enzyme in carbohydrate metabolism, hence inhibition of this enzyme activity will inhibit unwanted glucose metabolism thereby preventing blood glucose levels. The previous investigation by Sudatri et al. (2019), hypothesized that the extract activity might be due to flavonoids, but in the present investigation, it is proved that the anti-diabetic activity of EMCKG was totally due to the high content of triterpenoids.

Although reports are available regarding the anti-diabetic potential of *K. galanga* the majority of about rhizome extracts. As different solvent extracts possess different compositions, as well as essential oil composition, differs a lot from those of solvent extracts, hence, the present investigation gives a lot of impacts on the field of a new anti-diabetic agent search. Moreover, to fully validate as well as for the development of a successful anti-diabetic agent additional pre-clinical and clinical studies should be carried out.

### Anti-tyrosinase activity

The anti-tyrosinase activity of EMCKG revealed its strong skin whitening property superior to that of the standard. Earlier, *K. galanga* was reported for its skin disease preventive activities by Kumar (2020). Another study by Ko et al. (2014), on ethyl p-methoxy cinnamate crystals isolated from *K. galanga* chloroform fraction of ethanol extract, was reported for its ability to decrease melanin synthesis in B16F10 murine melanoma cells. But so far there is no report available regarding the skin-whitening activity of *K. galanga* rhizome essential oil. In the present investigation, ECMKG was found to compose of 13 different identified components with different area percentages.

An evaluation of the bioactivity of an individual component and a group of components is different. Recently an investigation showed ethyl cinnamate as a pure compound possesses strong skin whitening activity (Tepe & Ozaslan, 2020). In the present investigation, ethyl cinnamate composes 15.86% of the total essential oil. Hence, the strong anti-tyrosinase activity of the essential oil may be due to the presence of ethyl cinnamate in such a good quantity. So far, there is no report regarding *K. galanga* essential oil skin whitening activity available in the public domain making this the first-ever report. The findings also support the traditional use of the plant for various skin-related treatments. The strong anti-tyrosinase potential of the EMCKG makes way for the formulation and design of new skin whitening agents in the field of pharmaceuticals with easy availability, a cost-effective way.

### Antioxidant activity

The EMCKG showed more potent antioxidant properties than that of standard in the entire assay conducted for testing the antioxidant potential. A previous study on the rhizomes and leaves ethanolic extracts of *K. galanga* revealed that the antioxidant activity was found to be 77 mg ascorbic acid (AA)/100 g for leaves and 17 mg AA/100 g for rhizome respectively (Chan et al., 2008). In another study, the essential oil of (CP) conventionally propagated and (IVP) *in vitro* propagated plant of *K. galanga* was screened for DPPH scavenging activity. The study revealed the antioxidant activity was concentration-dependent with IC_50_ values, 6.6 (ascorbic acid), 26 (CP rhizome), and 19.5 µg/mL (IVP rhizome). Additionally, the hydrogen peroxide scavenging activity revealed a similar good antioxidant property against H_2_O_2_, with IC_50_ values of 29 (for essential oil samples of CP rhizome), 24.5 (for oil samples of IVP rhizome), and 21.5 µg/mL (for ascorbic acid) (Sahoo et al., 2014). A previous report by Ali et al. (2018) reported on the DPPH, ABTS and nitric oxide (NO) scavenging activity with IC_50_ values of 16.58, 8.24 and 38.16 μg/mL in DPPH, ABTS and no scavenging assays respectively. Chan & Lee (2019) reported that DPPH and ABTS radical scavenging activity in *Kaempferia galanga* increased in a concentration-dependent manner which is in a similar line to our findings. However, another study revealed weak anti-oxidant properties of *K. galanga* extracts (Mekseepralard, Kamkaen & Wilinson, 2010). Similarly, another study reported on the EMCKG that the antioxidant activity (DPPH assay) was inactive at a concentration of 100 µg/mL (Tewtrakul et al., 2005). The metal chelating activity on *Kaempferia galanga* was not previously reported by any findings thereof our result is the first report on antioxidant activity on ethyl p-methoxycinnamate rich *K. galanga* with the metal chelating assay. Our finding revealed that EMCKG has better antioxidant properties as compared to standard antioxidative agent ascorbic acid and further validates the ethnomedicinal use of *K. galanga*. The presence of high ethyl p-methoxycinnamate content may be the underlying reason for the high antioxidant activity of EMCKG. Additionally, as mentioned in the previous paragraph EMCKG was found to be an active inhibitor of the α-amylase enzyme, which property has a direct link with the anti-oxidant property of EMCKG. As there are evidence that ROS can react with insulin secreting β-cells thereby maximizing the chance of the development of diabetic condition (Fu et al., 2013). Hence a good antioxidant could be a good anti-diabetic agent. Thus, it can be concluded that EMCKG can be further analyzed for its use as a natural source of the antioxidative agent.

### Genotoxicity activity

Genotoxicity assay of EMCKG made it clear that at 1 µg/mL concentration the use of *K. galanga* essential oil is safe as it did not affect much the mitotic index and chromosomal aberration rates in the *Allium cepa* assay. So far, *K. galanga* crude rhizome extracts were tested for various toxicological studies such as contact toxicity, insecticidal effects, acute and sub-acute toxicities in rats, *etc*. (Kanjanapothi et al., 2004). So far there is no report available regarding the genotoxicity of *K. galanga* essential oil and this present report is the first of its kind. The present investigation showed that up to the concentration of 1 µg/mL the essential oil does not possess any genotoxic effect. Hence, this investigation result could give a safety tag of up to 1 µg/mL concentration of the essential oil. Therefore, in the near future formulation of the drug with potential effects could be designed by limiting the concentration up to the safety mark.

## Conclusions

The studied *K. galanga* rhizome essential oil was found to be rich in ethyl p-methoxycinnamate. Thus, an ethyl p-methoxycinnamate rich variety of *K. galanga* would be highly valuable and is required to meet the high industrial demand for raw materials for ethyl p-methoxycinnamate. The essential oil was found to possess a significant number of bioactivities like antioxidant, anti-inflammation, neurodegenerative disorders inhibitor, anti-diabetic, skin whitening as well as antimicrobial potential which further increases its pharmaceutical and industrial value in addition to validating its ethnopharmacological application. The essential oil also emerged as a very suitable source for raw materials in the formulation of anti-diabetic, skin whitening agents. From a toxicological point of view, the essential oil was found to be safe up to the concentration of 1 µg/mL. For the use of essential oil to formulate products or drugs it needs to go through further pre-clinical as well as deep clinical trials.

## Supplemental Information

10.7717/peerj.14606/supp-1 Supplemental Information 1 ANOVA and *Post hoc* analysis tables for EMCKG and standards.

## Additional Information and Declarations

### Data Availability

The following information was supplied regarding data availability: The raw data is available in the Supplemental File.
