# Supplementary material for: Novel ethyl p-methoxy cinnamate rich Kaempferia galanga (L.) essential oil and its pharmacological applications: special emphasis on anticholinesterase, anti-tyrosinase, α-amylase inhibitory, and genotoxic efficiencies
Source: PeerJ. 2023 Jan 9;11:e14606. doi: 10.7717/peerj.14606 (PMC9835694; doi:10.7717/peerj.14606)
Supplement: Supplemental Information 1 [file peerj-11-14606-s001.docx]

**Novel ethyl p-methoxy cinnamate rich *Kaempferia galanga* Linn., essential oil and its pharmacological applications: special emphasis on anticholinesterase, anti-tyrosinase, α-amylase inhibitory, and genotoxic efficiencies**

Twahira Begum^a.b^, Roktim Gogoi^a,b^, Neelav Sarma^a,b^, Sudin Kumar Pandey^a,b^ and Mohan Lal^b^*

^a^AcSIR-Academy of Scientific and Innovative Research, Ghaziabad, Uttar Pradesh, 201002 India,

^b^Agrotechnology and Rural Development Division

CSIR-North East Institute of Science and Technology (NEIST), Jorhat, Assam-785006, India

*Corresponding Author Email id: drmohanlal80@gmail.com

**Supplementary data:**

**Table S1:** Analysis of variance for bioactivity

Table S1.1 ANOVA for antioxidant activity of the essential oil performed by different methods

| *Source of Variation* | *SS* | *df* | *MS* | *F* | *P-value* | *F crit* |
| --- | --- | --- | --- | --- | --- | --- |
| Treatment | 143.9133 | 2 | 71.95665 | 0.365412 | 0.701372 | 3.885294 |
| Error | 2363.03 | 12 | 196.9191 |  |  |  |
| Total | 2506.943 | 14 |  |  |  |  |

*SS= sum of squares, df= degree of freedom, MS= mean square

Table S1.2 ANOVA for antioxidant activity of standards used performed by different methods

| *Source of Variation* | *SS* | *df* | *MS* | *F* | *P-value* | *F crit* |
| --- | --- | --- | --- | --- | --- | --- |
| Treatment | 412.7137 | 2 | 206.3568 | 0.891398 | 0.430727 | 3.68232 |
| Error | 3472.47 | 15 | 231.498 |  |  |  |
| Total | 3885.184 | 17 |  |  |  |  |

*SS= sum of squares, df= degree of freedom, MS= mean square

Table S1.3 ANOVA for anti-inflammatory activity of the essential oil performed by different methods

| *Source of Variation* | *SS* | *df* | *MS* | *F* | *P-value* | *F crit* |
| --- | --- | --- | --- | --- | --- | --- |
| Treatment | 1071.189 | 1 | 1071.189 | 3.105093 | 0.108529 | 4.964603 |
| Error | 3449.781 | 10 | 344.9781 |  |  |  |
| Total | 4520.97 | 11 |  |  |  |  |

*SS= sum of squares, df= degree of freedom, MS= mean square

Table S1.4 ANOVA for anti-inflammatory activity of standards used performed by different methods

| *Source of Variation* | *SS* | *df* | *MS* | *F* | *P-value* | *F crit* |
| --- | --- | --- | --- | --- | --- | --- |
| Treatment | 368.0407 | 1 | 368.0407 | 0.794438 | 0.393702 | 4.964603 |
| Error | 4632.718 | 10 | 463.2718 |  |  |  |
| Total | 5000.759 | 11 |  |  |  |  |

*SS= sum of squares, df= degree of freedom, MS= mean square

Table S1.5 ANOVA for anticholinesterase activity for results between standard and essential oil

| *Source of Variation* | *SS* | *df* | *MS* | *F* | *P-value* | *F crit* |
| --- | --- | --- | --- | --- | --- | --- |
| Treatment | 1280.369 | 1 | 1280.369 | 5.783289 | 0.037008 | 4.964603 |
| Error | 2213.911 | 10 | 221.3911 |  |  |  |
| Total | 3494.281 | 11 |  |  |  |  |

*SS= sum of squares, df= degree of freedom, MS= mean square

Table S1.6 ANOVA for anti-diabetic activity for results between standard and essential oil

| *Source of Variation* | *SS* | *df* | *MS* | *F* | *P-value* | *F crit* |
| --- | --- | --- | --- | --- | --- | --- |
| Treatment | 53.91867 | 1 | 53.91867 | 0.10887 | 0.748242 | 4.964603 |
| Error | 4952.562 | 10 | 495.2562 |  |  |  |
| Total | 5006.481 | 11 |  |  |  |  |

*SS= sum of squares, df= degree of freedom, MS= mean square

Table S1.7 ANOVA for anti-tyrosinase activity for results between standard and essential oil

| *Source of Variation* | *SS* | *df* | *MS* | *F* | *P-value* | *F crit* |
| --- | --- | --- | --- | --- | --- | --- |
| Treatment | 154.411 | 1 | 154.411 | 0.439994 | 0.531769 | 5.987378 |
| Error | 2105.635 | 6 | 350.9391 |  |  |  |
| Total | 2260.046 | 7 |  |  |  |  |

*SS= sum of squares, df= degree of freedom, MS= mean square

**Table S2:** Post hoc analysis table for anticholinesterase activity for results between standard and essential oil

| Treatments  pair | Tukey HSD  Q statistic | Tukey HSD  p-value | Tukey HSD  inferfence |
| --- | --- | --- | --- |
| A vs B | 3.4010 | 0.0370104 | * p<0.05 |

**Table S3:** Raw data used for bioactivity

| Ascorbic acid DPPH activity | | | |
| --- | --- | --- | --- |
| conc | % inhibition1 | % inhibition2 | %inhibition3 |
| 5 | 20.76 | 21.86 | 19.96 |
| 10 | 30.25 | 31.16 | 28.48 |
| 15 | 41.34 | 42.77 | 39.62 |
| 20 | 46.98 | 48.02 | 47.65 |
| 25 | 56.82 | 58.36 | 57.82 |
| 30 | 63.02 | 64.28 | 63.68 |

conc= concentration (µg/mL)

| MCKG DPPH | | | |
| --- | --- | --- | --- |
| Conc (µg/mL) | % inhibition1 | % inhibition2 | %inhibition3 |
| 5 | 24.36 | 23.44 | 25.21 |
| 10 | 38.43 | 39.3 | 40.08 |
| 15 | 48.47 | 47.64 | 48.12 |
| 20 | 58.52 | 56.24 | 57.76 |
| 25 | 70.12 | 69.33 | 70.28 |
| 30 | 87.42 | 86.28 | 88.26 |

conc= concentration µg/mL

| Ascorbic acid ABTS result | | | |
| --- | --- | --- | --- |
| Conc (µg/mL) | % inhibition1 | %inhibition2 | %inhibition3 |
| 5 | 19.85 | 20.44 | 21.68 |
| 10 | 28.65 | 31.26 | 30.42 |
| 15 | 39.33 | 41.05 | 40.73 |
| 20 | 46.74 | 48.63 | 47.88 |
| 25 | 52.38 | 53.66 | 54.14 |
| 30 | 68.84 | 66.52 | 69.38 |

conc= concentration µg/mL

| MCKG ABTS activity | | | |
| --- | --- | --- | --- |
| Conc (µg/mL) | % inhibition1 | % inhibition2 | %inhibition3 |
| 5 | 33.18 | 32.5 | 31.27 |
| 10 | 35.32 | 36.28 | 34.55 |
| 15 | 52.37 | 51.3 | 50.66 |
| 20 | 53.42 | 54.22 | 55.26 |
| 25 | 62.71 | 60.18 | 61.82 |

conc= concentration µg/mL

| EDTA metal chelating activity | | | |
| --- | --- | --- | --- |
| Conc (µg/mL) | % inhibition1 | %inhibition2 | %inhibition3 |
| 5 | 18.24 | 16.5 | 22.06 |
| 10 | 22.15 | 19.74 | 24.5 |
| 15 | 30.17 | 28.7 | 34.64 |
| 20 | 36.2 | 34.42 | 35.48 |
| 25 | 41.85 | 41.16 | 41.62 |
| 30 | 51.34 | 48.26 | 52.34 |

conc= concentration µg/mL

| MCKG metal chelating | | | |
| --- | --- | --- | --- |
| Conc (µg/mL) | % inhibition1 | %inhibition2 | %inhibition3 |
| 5 | 26.11 | 25.34 | 20.3 |
| 10 | 32.06 | 30.08 | 33.45 |
| 15 | 41.56 | 42.44 | 46.52 |
| 20 | 50.32 | 53.6 | 55.24 |
| 25 | 58.1 | 60.25 | 62.82 |
| 30 | 66.29 | 68.5 | 70.08 |

conc= concentration µg/mL

| Sodium diclofenac albumin denaturation | | | |
| --- | --- | --- | --- |
| Conc (µg/mL) | % inhibition1 | % inhibition2 | %inhibition3 |
| 10 | 28.45 | 27.96 | 29.04 |
| 15 | 36.25 | 35.88 | 37.16 |
| 20 | 50.36 | 49.74 | 51.44 |
| 25 | 69.38 | 67.99 | 70.26 |
| 30 | 79.33 | 75.82 | 76.48 |
| 35 | 88.21 | 87.78 | 88.25 |

conc= concentration µg/mL

| MCKG Protein denaturation | | | |
| --- | --- | --- | --- |
| Conc (µg/mL) | % inhibition1 | % inhibition2 | %inhibition3 |
| 10 | 56.36 | 57.12 | 56.48 |
| 15 | 67.85 | 68.24 | 65.18 |
| 20 | 84.6 | 86.56 | 85.92 |
| 25 | 88.02 | 88.4 | 87.28 |
| 30 | 91.94 | 91.42 | 90.51 |
| 35 | 96.86 | 97.5 | 96.88 |

conc= concentration µg/mL

| Sodium diclofenac protease inhibitory result | | | |
| --- | --- | --- | --- |
| Conc (µg/mL) | % inhibition1 | % inhibition2 | %inhibition3 |
| 10 | 24.52 | 20.46 | 23.82 |
| 15 | 30.63 | 28.75 | 29.28 |
| 20 | 41.66 | 38.55 | 38.96 |
| 25 | 58.2 | 57.32 | 58.39 |
| 30 | 65.75 | 62.48 | 64.08 |
| 35 | 70.84 | 66.78 | 69.94 |

conc= concentration µg/mL

| MCKG protease inhibitory results | | | |
| --- | --- | --- | --- |
| Conc (µg/mL) | % inhibition1 | % inhibition2 | %inhibition3 |
| 10 | 31.64 | 30.24 | 32.19 |
| 15 | 48.42 | 47.56 | 48.64 |
| 20 | 56.88 | 54.42 | 57.81 |
| 25 | 68.23 | 66.85 | 69.74 |
| 30 | 79.16 | 76.97 | 80.22 |
| 35 | 90.47 | 88.52 | 89.03 |

conc= concentration µg/mL

| Kojic acid tyrosinase inhibitory result | | | |
| --- | --- | --- | --- |
| Conc (µg/mL) | % inhibition1 | %inhibition2 | %inhibition3 |
| 0 | 0 | 0 | 0 |
| 5 | 14.28 | 13.55 | 14.86 |
| 10 | 26.14 | 27.16 | 26.5 |
| 15 | 39.22 | 42.07 | 40.66 |
| 20 | 58.1 | 57.48 | 58.94 |

conc= concentration µg/mL

| MCKG tyrosinase inhibitory result | | | |
| --- | --- | --- | --- |
| Conc (µg/mL) | % inhibition1 | %inhibition2 | %inhibition3 |
| 0 | 0 | 0 | 0 |
| 5 | 22.18 | 20.46 | 21.73 |
| 10 | 36.26 | 35.22 | 35.68 |
| 15 | 55.92 | 54.88 | 56.24 |
| 20 | 63.08 | 60.11 | 62.64 |

conc= concentration µg/mL

| Galanthamine AChE activity results | | | |
| --- | --- | --- | --- |
| Conc (µg/mL) | %inhibition1 | %inhibition2 | %inhibition3 |
| 10 | 12.8 | 13.24 | 12.68 |
| 15 | 27.32 | 28.46 | 26.85 |
| 20 | 37.89 | 36.08 | 35.62 |
| 25 | 48.55 | 49.21 | 48.74 |
| 30 | 54.63 | 55.02 | 49.51 |
| 35 | 65.22 | 65.22 | 64.82 |

conc= concentration µg/mL

| MCKG AChE activity results | | | |
| --- | --- | --- | --- |
| Conc (µg/mL) | % inhibition1 | %inhibition2 | %inhibition3 |
| 10 | 23.73 | 24.11 | 23.68 |
| 15 | 31.22 | 32.46 | 30.79 |
| 20 | 47.09 | 46.11 | 45.44 |
| 25 | 59.32 | 60.2 | 61.24 |
| 30 | 68.88 | 69.84 | 68.42 |
| 35 | 76.25 | 76.25 | 77.1 |

conc= concentration µg/mL

| Acarbose alpha amylase inhibitory result | | | |
| --- | --- | --- | --- |
| Conc (µg/mL) | % inhibition1 | %inhibition2 | %inhibition3 |
| 5 | 14.19 | 15.22 | 15.03 |
| 10 | 22.84 | 23.72 | 22.96 |
| 15 | 38.02 | 39.26 | 40.1 |
| 20 | 51.32 | 53.22 | 52.46 |
| 25 | 59.86 | 60.54 | 58.82 |
| 30 | 70.42 | 71.26 | 70.72 |

conc= concentration µg/mL

| MCKG amylase inhibitor results | | | |
| --- | --- | --- | --- |
| Conc (µg/mL) | % inhibition1 | %inhibition2 | %inhibition3 |
| 5 | 18.28 | 17.2 | 18.66 |
| 10 | 27.44 | 25.86 | 27.52 |
| 15 | 42.05 | 39.77 | 41.5 |
| 20 | 58.11 | 55.18 | 56.23 |
| 25 | 67.4 | 64.3 | 66.84 |
| 30 | 78.24 | 75.65 | 76.04 |

conc= concentration µg/mL
